# Exploring Leptin Antagonism in Ophthalmic Cell Models

**DOI:** 10.1371/journal.pone.0076437

**Published:** 2013-10-03

**Authors:** Laura Scolaro, Cristina Parrino, Roberta Coroniti, Laszlo Otvos, Eva Surmacz

**Affiliations:** 1 Sbarro Institute for Cancer Research and Molecular Medicine, Biotechnology Center, Temple University, Philadelphia, Pennsylvania, United States of America; 2 Department of Biology, Temple University, Philadelphia, Pennsylvania, United States of America; Virginia Commonwealth University, United States of America

## Abstract

**Background:**

Emerging evidence suggests that angiogenic and pro-inflammatory cytokine leptin might be implicated in ocular neovascularization. However, the potential of inhibiting leptin function in ophthalmic cells has never been explored. Here we assessed mitogenic, angiogenic, and signaling leptin activities in retinal and corneal endothelial cells and examined the capability of a specific leptin receptor (ObR) antagonist, Allo-aca, to inhibit these functions.

**Methods and Results:**

The experiments were carried out in monkey retinal (RF/6A) and bovine corneal (BCE) endothelial cells. Leptin at 50-250 ng/mL stimulated the growth of both cell lines in a dose-dependent manner. The maximal mitogenic response (35±7 and 27±3% in RF6A and BCE cells, respectively) was noted at 24 h of 250 ng/mL leptin treatments. Leptin-dependent proliferation was reduced to base levels with 10 and 100 nM Allo-aca in BCE and RF6A cells, respectively. In both cell lines, leptin promoted angiogenic responses, with the maximal increase in tube formation (163±10 and 133±8% in RF6A and BCE cultures, respectively) observed under a 250 ng/mL leptin treatment for 3 h. Furthermore, in both cell lines 250 ng/mL leptin modulated the activity or expression of several signaling molecules involved in proliferation, inflammatory activity and angiogenesis, such as STAT3, Akt, and ERK1/2, COX2, and NFκB. In both cell lines, leptin-induced angiogenic and signaling responses were significantly inhibited with 100 nM Allo-aca. We also found that leptin increased its own mRNA and protein expression in both cell lines, and this autocrine effect was abolished by 100-250 nM Allo-aca.

**Conclusions:**

Our data provide new insights into the role of leptin in ocular endothelial cells and represent the first original report on targeting ObR in ophthalmic cell models.

## Introduction

Angiogenesis plays a central role in adult tissue homeostasis and is also responsible for several pathological conditions, including those affecting the eye [1,2]. Ocular neovascularization is a pathological hallmark of some forms of vision-threatening complications, including proliferative diabetic retinopathy (PDR), age related macular degeneration (AMD) and corneal pathologies [2-5].

The complex pathophysiology of ocular neovascularization reflects impairment of metabolic, endocrine and hematologic systems, which leads to the development of local imbalance between pro-angiogenic/inflammatory factors and their modulators [2,4]. The overexpression of vascular endothelial growth factor (VEGF) is thought to be the leading cause of abnormal vessel formation in the eye. However, several other activators of angiogenesis such as platelet-derived growth factor, basic fibroblast growth factor (bFGF), hepatocyte growth factor, interleukins 1a, 6 and 8, and leptin have also been implicated [6]. Many of these factors act through upregulation of VEGF synthesis but their direct involvement remains largely unclear [1,6]. At present, VEGF targeting drugs (i.e., ranibizumab, a modified anti-VEGF antibody and aflibercept, a VEGF trap fusion protein) are approved for the treatment of wet AMD and diabetic macular edema (DME), and experimentally used for other eye diseases, e.g., PDR [7]. However, adverse effects (systemic and ocular) and development of resistance to the treatment have been noted with long-term use. Thus, targeting pro-angiogenic factors other than VEGF could be prove to be an effective alternative or complementary therapy for pathological neovascularization in the eye [4,6-9].

This study focuses on molecular targeting of pro-angiogenic action of leptin in retinal and corneal cell models. Leptin, a pluripotent cytokine has been first described as an adipocyte-derived hormone that regulates energy expenditure and food intake via hypothalamic effects [10,11]. Later studies proved that leptin is expressed in different peripheral organs and tissues and is involved in multiple physiological and pathological processes, such as immune response, hematopoiesis, fertility, bone remodeling, cardiovascular disease, type 2 diabetes, and cancer [12-16].

Of special interest is the ability of leptin to regulate normal and abnormal angiogenesis. The leptin receptor (ObR) was detected in vascular endothelial cells and studies in vitro demonstrated that leptin can induce angiogenic differentiation, migration and proliferation in endothelial cells. Most of these studies were carried out using human umbilical vein endothelial cells (HUVEC) or aortic endothelial cells [17-23]; only one study involved retinal endothelial cells [24].

Leptin exerts its effects through multiple intracellular signals, including the Janus kinase 2/signal transducer and activator of transcription (JAK2/STAT3), Ras/extracellular signal-regulated kinase 1/2 (Ras/ERK1/2), phosphoinositide 3 kinase/protein kinase B/glycogen synthase kinase 3 (PI-3K/Akt/GSK3) as well as pro-inflammatory cyclooxygenase 2 (COX2) and nuclear factor kappa B (NFκB) pathways [21,25-28]. In HUVEC, the use of specific inhibitors suggested that leptin-mediated angiogenesis depends on ObR crosstalk with VEGFR2 and is mediated through a functional axis involving p38^MAPK^, Akt/PI3K/Akt, and COX2 [21]. Interestingly, some studies show that leptin-induced angiogenesis in HUVEC can be partially reduced with VEGFR inhibitor [21], while others did not observe such effects [29], suggesting independent leptin action.

A few recent studies addressed the role of leptin in ophthalmic experimental models. ObR was detected in primary porcine retinal endothelial cells and leptin treatment stimulated STAT3 phosphorylation and induced VEGF mRNA expression in this model [24]. In a corneal angiogenic assay, leptin stimulated vessel formation synergistically with FGF [23]. However, leptin was not able to induce neovascularization in corneas from fa/fa Zucker rats that lack functional ObR [20]. In mouse models, transgenic overexpression of the leptin gene (*ob*) potentiated ischemia-induced retinal neovascularization, while leptin deficiency due to ob inactivation, significantly reduced ocular angiogenesis. Leptin action in ob transgenic mouse model was mediated, at least in part, through increased VEGF expression [24]. Noteworthy, alkali-induced corneal neovascularization in normal mice was associated with leptin and VEGF overexpression in the regions of new vessels formation [30].

While experimental data suggested leptin involvement in ocular neovascularization, relevant clinical reports are scarce and occasionally conflicting. Gariano et al. demonstrated that in a group of 48 patients with proliferative diabetic retinopathy (PDR) or retinal detachment (RD), intravitreous leptin levels were significantly elevated relative to leptin expression in the eyes of patients with other ocular diseases [31]. In addition, the study suggested that locally produced leptin, not simply leptin derived form circulation, could be involved in the pathogenesis of PDR and RD [31]. Similarly, a small study confirmed higher vitreous leptin levels in PDR relative to other retinopathies [32]. On the other hand, other preliminary analysis involving 25 patients with PDR demonstrated that intravitreous leptin was not directly associated with the disease [33].

Until present, blocking leptin signals in experimental ophthalmic models has not been attempted. We recently generated peptide-based compounds that interfere with leptin/ObR binding and downstream signaling [15,34]. The lead ObR antagonist, Allo-aca, is a 9 residue peptidomimetic that inhibits leptin-induced proliferation and signaling at pM-low nM concentrations in vitro and exhibits anti-neoplastic and anti-inflammatory activities in vivo at 0.1-0.5 mg/kg/day doses [35-37]. The efficacy of Allo-aca in endothelial cells has never been addressed and is explored here in retinal and corneal cell models.

## Methods

### Reagents

The ObR antagonist, Allo-aca, is a short leptin-based peptidomimetic (H-alloThr-Glu-Nva-Val-Ala-Leu-Ser-Arg-Aca-NH_2_) whose sequence is based on leptin/ObR binding site III. The design, development and efficacy of Allo-aca in vitro and in vivo have been reported by us before [35,38-40]. An unrelated peptide Chex1-Arg20: H-Chex-Arg-Pro-Asp-Lys-Pro-Arg-Pro-Tyr-Leu-Pro-Arg-Pro-Arg-Pro-Pro-Arg-Pro-Val-Arg-NH_2_ was used as control [37].

Leptin (human recombinant) and VEGF (human recombinant, VEGF 165) were purchased from R&D Systems (Minneapolis, MN).

### Cell lines and growth conditions

Monkey endothelial retinal cells (RF/6A) and bovine endothelial corneal cells (BCE) were purchased from the American Type Culture Collection (Rockville, MD, USA). RF/6A cells were grown in Minimum Essential Medium (MEM) containing 1 g/L glucose, 10% fetal bovine serum (FBS) and 1% penicillin/streptomycin (P/S). BCE cells were grown in Dulbecco’s modified Eagle’s medium (DMEM) containing 4.5 g/L glucose, L-glutamine, sodium pyruvate, 10% FBS and 1% P/S. All culture reagents and media were purchased from Cellgro (Cellgro, Herndon, VA, USA).

### Proliferation assay

The cells (2-5^th^ passage) were plated in 24 well plates at concentrations 8 x10^4^ and 1x10^5^ cells/well for RF/6A and BCE cells, respectively. At 70% confluence, the cells were shifted to serum-free medium (SFM: MEM or DMEM with 10 µM FeSO_4_, 0.5% bovine serum albumin, 1% FBS, 1% P/S) for 24 h and then treated with 50-500 ng/mL of leptin (R&D) for 24 or 48 h. The effects of 10-500 nM Allo-aca on leptin mitogenic activity were tested following the analogous protocol. Cell numbers before and after treatments were determined by direct counting with trypan blue exclusion. All assays were done in triplicate and repeated 3-6 times. The percentage decrease/increase in cell number vs. control SFM was calculated and expressed as mean ± standard deviation (SD).

### Intracellular signaling

To assess short-term effects of leptin in RF/6A and BCE cells, 70% confluent cell cultures at 4^th^ or 5^th^ passage were shifted to SFM for 24 h and then treated with 250 ng/mL leptin for 15 or 30 min, or left untreated. To test the effects of Allo-aca on leptin signaling, the cells were pretreated with the antagonist at 10-100 nM for 1 h before leptin addition. The long-term effects of leptin and Allo-aca were determined at 6, 12, and 24 h post treatment. Next, the cells were lysed and total cellular proteins were obtained as described previously [41]. The expression of ObR and downstream signaling molecules was evaluated by Western Blot (WB) in 50-100 µg of total proteins. The following primary antibodies (Abs) from Cell Signaling Technology (Danvers, MA) were used: for phospho-Akt, Akt Ser473 pAb, 1:500; for total Akt, Akt pAb, 1:1000; for phospho-STAT3, STAT3 Tyr705, D3A7 mAb, 1:500; for total STAT3, STAT3 79D7 mAb, 1:500; for phospho-ERK1/2, p44/42 mitogen-activated protein kinase (MAPK; ERK1/2) pAb Thr202/Tyr204, 1:1000; for total ERK1/2, p44/42 MAPK pAb, 1:1000; for total COX2, COX2 pAb, 1:250. The following primary Abs from Santa Cruz were used: for ObR, H-300 pAb, 1:500; for NFκB, for NFκB p65A, pAb 1:500; for β-actin, Actin I-19 pAb, 1:500.The intensity of bands corresponding to studied proteins was measured using ImageJ program as described before [22].

### Angiogenic assay

The ability of cells to migrate and organize into enclosed spaces (ES) on Matrigel was carried out as described in detail previously [22]. Briefly, the cells at 4^th^ or 5^th^ passage were shifted to SFM for 24 h. Next, the cells at 1x10^4^ (RF/6A) and 2x10^4^ (BCE) were suspended in 200 µl SFM containing either leptin at different concentrations, leptin plus Allo-aca at different concentrations, Allo-aca alone, control unrelated peptide, or VEGF at different concentrations. SFM alone was used as a negative control. The mixtures were seeded in 96 well plates covered with polymerized growth factor-reduced Matrigel matrix (BD, Franklin Lakes, NJ), incubated for 3 h at 37°C and photographed using Olympus 1x81 phase-contrast microscope at 3.2 x magnification and Metamorph 7.5 program. The number of ES in the whole photographed area (representing central 70% of the well) was scored by two observers. Each experiment was done in triplicate and repeated at least 3 times. The mean number of ES ± SD was determined for each condition.

### Quantitative Real Time PCR (qRT-PCR)

RF/6A and BCE cells at 70% confluence were shifted to SFM for 24 h and then treated with 250 ng/mL leptin for 3, 6, 24 h. To test the effects of Allo-aca, the cultures were pretreated 10-250 nM Allo-aca for 1 h. RNA was isolated using Trizol Reagent (Life Technologies, Grand Island, NY) according to manufacturer’s instructions. A total of 4 µg of RNA was reverse transcribed in 20 µL of reaction volume using the High-Capacity cDNA Kit (Life Technologies). Four µL of the RT product were used to amplify leptin sequences using TaqMan probes Bt03211909_m1 for bovine leptin and Rh02788316_m1 for monkey leptin (Life Technologies). To normalize qRT-PCR results, parallel reactions were run on each sample for β-actin using a TaqMan probe (Life Technologies). The levels of target mRNA relative to β-actin mRNA were determined using a comparative CT method, as suggested by the manufacturer (Life Technologies). All reactions were done in triplicate and an average CT value (±SD) for all RNAs was calculated. The individual experiments were repeated at least 3 times.

### Immunofluorescence

Leptin protein was detected in RF/6A and BCE cells by immunofluorescence (IF), as described by us before [42]. In short, 1x10⁵ cells were plated on sterile glass cover slips in normal growth medium. After 24 h, the cells were synchronized in SFM for 24 h and then treated with 250 ng/mL leptin in the presence or absence of 100 or 250 nM Allo-aca for 24 h. Next, the cells were washed with PBS, fixed for 10 minutes in methanol at -20°C, and permeabilized in 0.2 Triton X-100% for 5 min at room temperature. Leptin expression was detected using pAb A-20 (1:25 dilution; 2 h) and goat anti-rabbit IgG-FITC (1:1000 plus 1.5% blocking goat serum; 1 h). In control experiments, primary Abs were replaced by non-immune serum. Following staining, the coverslips were mounted using UltraCruz Mounting Medium containing 1.5 µg/mL of 4’,6-diamidino-2-phenylindole (DAPI) to allow visualization of cell nuclei. All Abs and other reagents were purchased from Santa Cruz Biotechnology. The expression of leptin under different conditions was assessed using Olympus 1x81 phase-contrast microscope at 2 x magnification and Metamorph 7.5 program. The percentage of positive cells was determined in 10 visual fields.

### Statistical analysis

All experiments were done at least in triplicates and data analyzed by Student’s t-test.

Differences with p values of ≤ 0.05 were considered significant.

## Results

### ObR antagonist, Allo-aca, inhibits leptin growth effects in RF/6A and BCE cells

**Figure 1:**
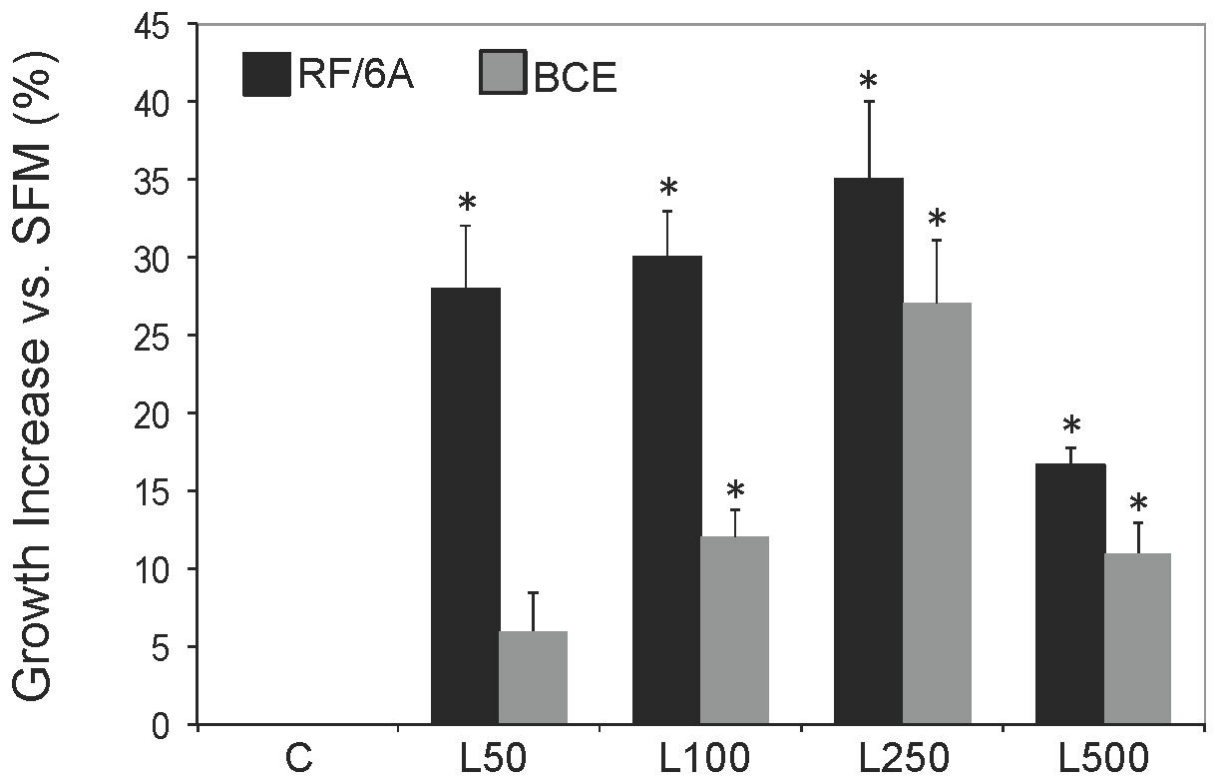
Leptin growth response in RF/6A retinal and BCE bovine corneal endothelial cells. RF/6A and BCE cells were synchronized in SFM and stimulated with 50-250 ng/mL leptin (L) for 24 h. The % increase (±SD) in cell number vs. untreated control (C=SFM) is shown. Asterisks indicate significant (p≤0.05) differences vs. SFM.

We first tested leptin time and dose-responses in RF/6A retinal and BCE corneal cells. Leptin was used at 50-500 ng/mL concentrations and cells were treated for 24-72 h. The maximal growth responses in both cell lines were observed at 24 h stimulation (data not shown), thus this time point was used in further experiments. In RF/6A and BCE cells, a 24 h leptin treatment induced cell growth in a dose-dependent manner at 50-250 ng/mL. The maximal mitogenic effect, i.e., ~35% and 27%, in RF6A and BCE cells, respectively, was observed with 250 ng/mL, while the response declined under 500 ng/mL leptin concentrations (Figure 1).

**Figure 2:**
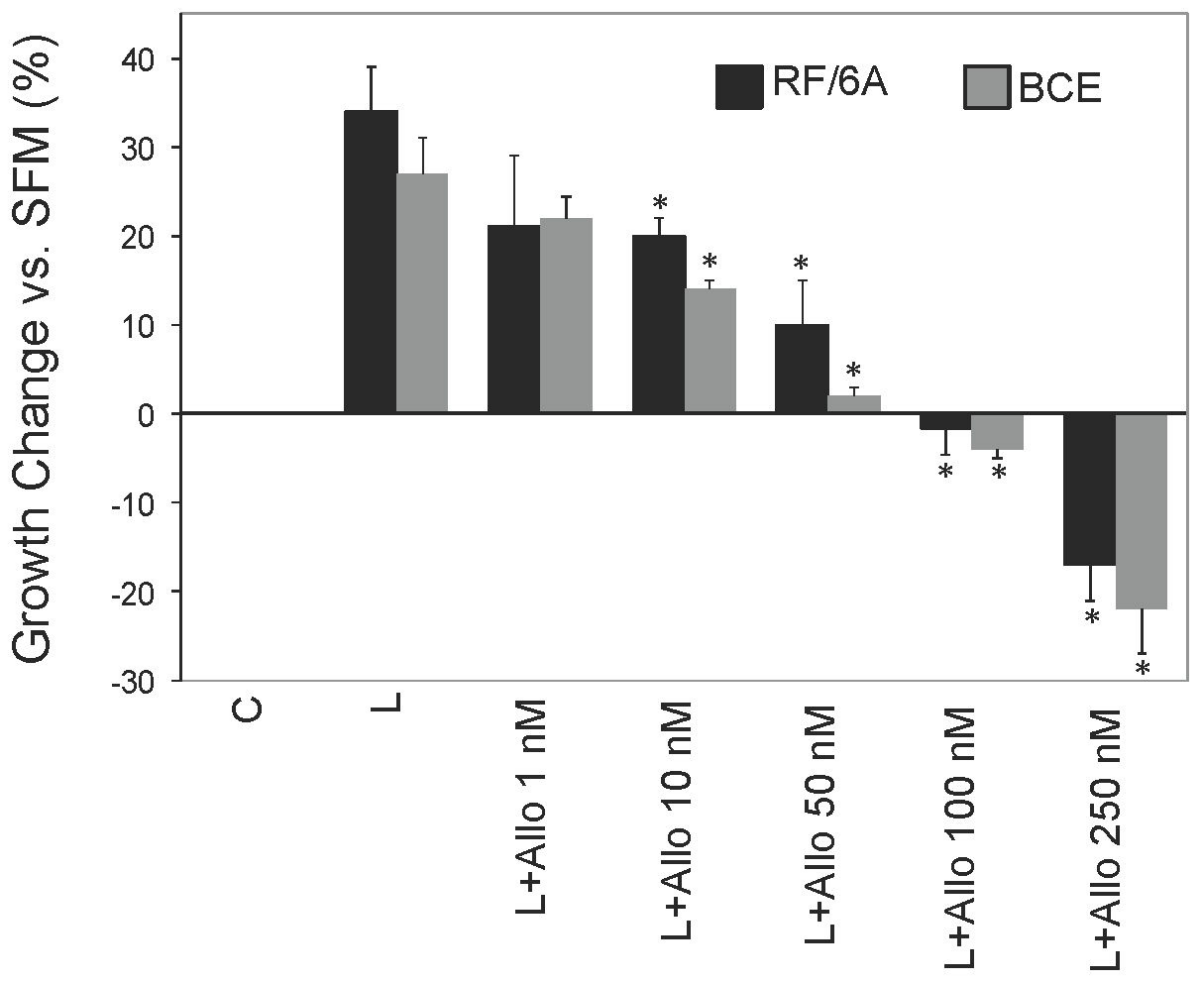
Effects of Allo-aca on leptin-dependent proliferation in RF/6A and BCE cells. RF/6A and BCE cells were synchronized in SFM and stimulated with 250 ng/mL leptin (L) in the presence or absence of 1-250 nM Allo-aca (Allo) for 24 h. The % increase/decrease (±SD) in cell number vs. untreated control (C=SFM) is shown. Asterisks indicate significant (p≤0.05) differences vs. leptin.

In both cell lines, the treatment with 10-250 nM Allo-aca significantly reduced mitogenic activity of 250 ng/mL leptin. A complete inhibition was observed with 100 nM Allo-aca concentrations, while at 250 nM, the peptide reduced cell proliferation ~20% below base levels (Figure 2), possibly by inhibiting endogenous leptin expression (see below). When added alone, Allo-aca did not produce any significant cytostatic or cytotoxic effects at 1-100 nM concentrations. In RF/6A cells, a weak (9%) agonist activity of 250 nM Allo-aca was detected. This effect, however, was not noted in BCE cells. A control peptide was inactive in this assay (data not shown).

### ObR antagonist blocks leptin-induced angiogenic effects in RF/6A and BCE cells

**Figure 3:**
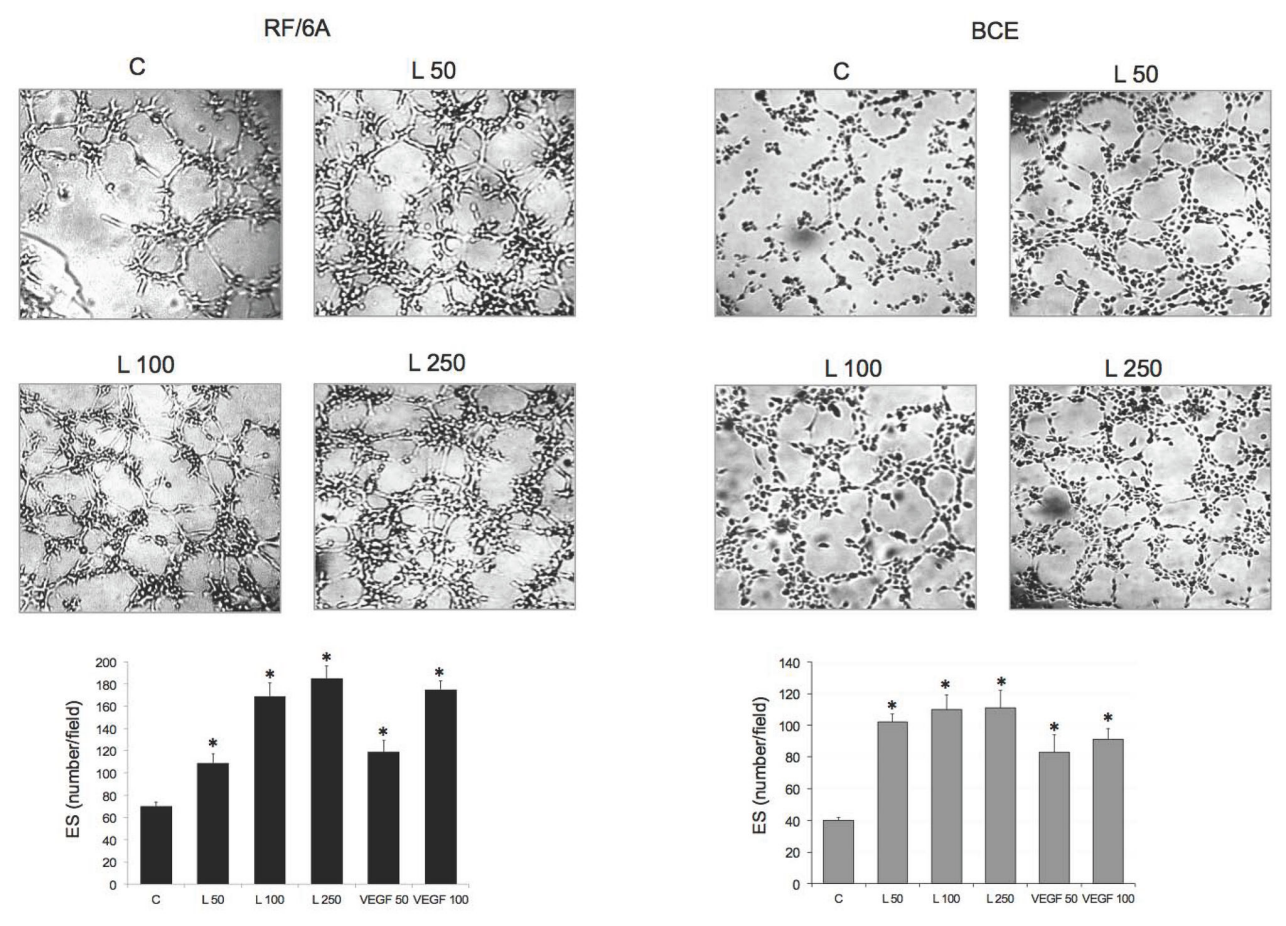
Effects of leptin on angiogenic response in RF/6A and BCE cells. The assays were carried out as described in Materials and Methods. The photographs represent ES formation in RF/6A and BCE cultures under different treatments: SFM (C), 50-250 ng/mL leptin (L), 50 and 100 ng/mL VEGF (central field of the well at 5x magnification is shown). The graph shows the number of ES (±SD) per visual field. Asterisks indicate significant (p≤0.05) differences vs. SFM.

In both cell lines, leptin increased ES formation at 50-250 ng/mL concentrations. The maximal stimulation of tube formation (by ~3.0- and 2.75-fold in RF/6A and BCE cells, respectively) was noted under the 250 ng/mL leptin treatment for 3 h (Figure 3). This effect was comparable to that of 100 ng/mL VEGF in RF6A and 200 ng/mL VEGF in BCE cells (Figure 3 and data not shown).

**Figure 4:**
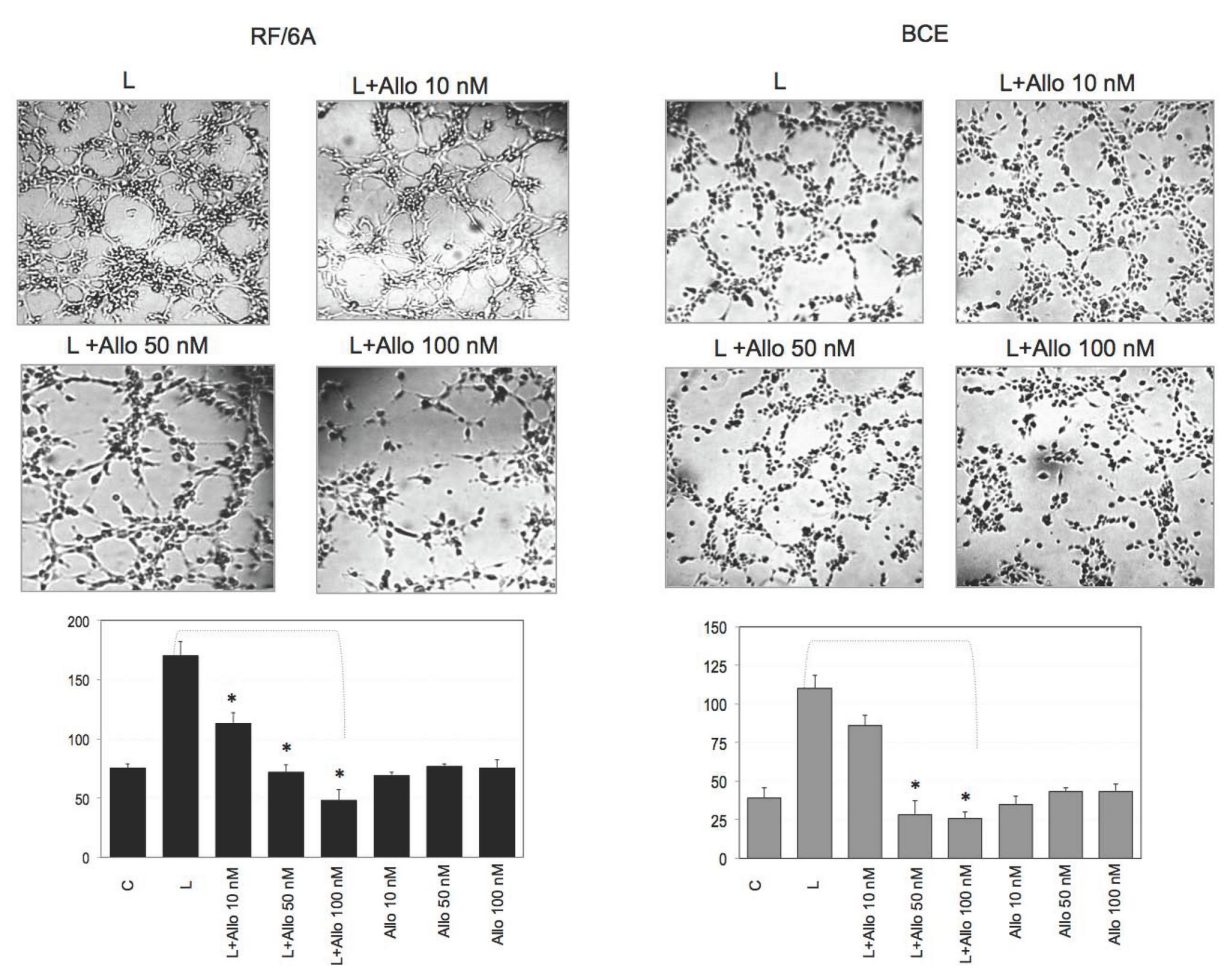
Effects of Allo-aca on leptin angiogenic activity in RF/6A and BCE cells. The assays were carried out as described in Materials and Methods. Untreated cells (C=SFM) and cells treated with 250 leptin (L) alone, L+ 1-100 nM Allo-aca (Allo), or 10-100 nM Allo-aca. The photographs represent ES formation under different treatments in RF/6A and BCE cells (central field of the well at 5x magnification is shown). The graph shows the number of ES (±SD) per visual field. Asterisks indicate significant (p≤0.05) differences vs. leptin.

The angiogenic activity of 250 ng/mL leptin was totally blocked by Allo-aca at 50-100 nM concentrations in both cell lines (Figure 4). Allo-aca alone did not affect tube formation up to 100 nM concentrations, and a control peptide was totally neutral in angiogenesis assays (Figure 4 and data not shown).

### ObR antagonist inhibits several leptin-dependent acute and long-term intracellular responses in RF/6A and BCE cells

**Figure 5:**
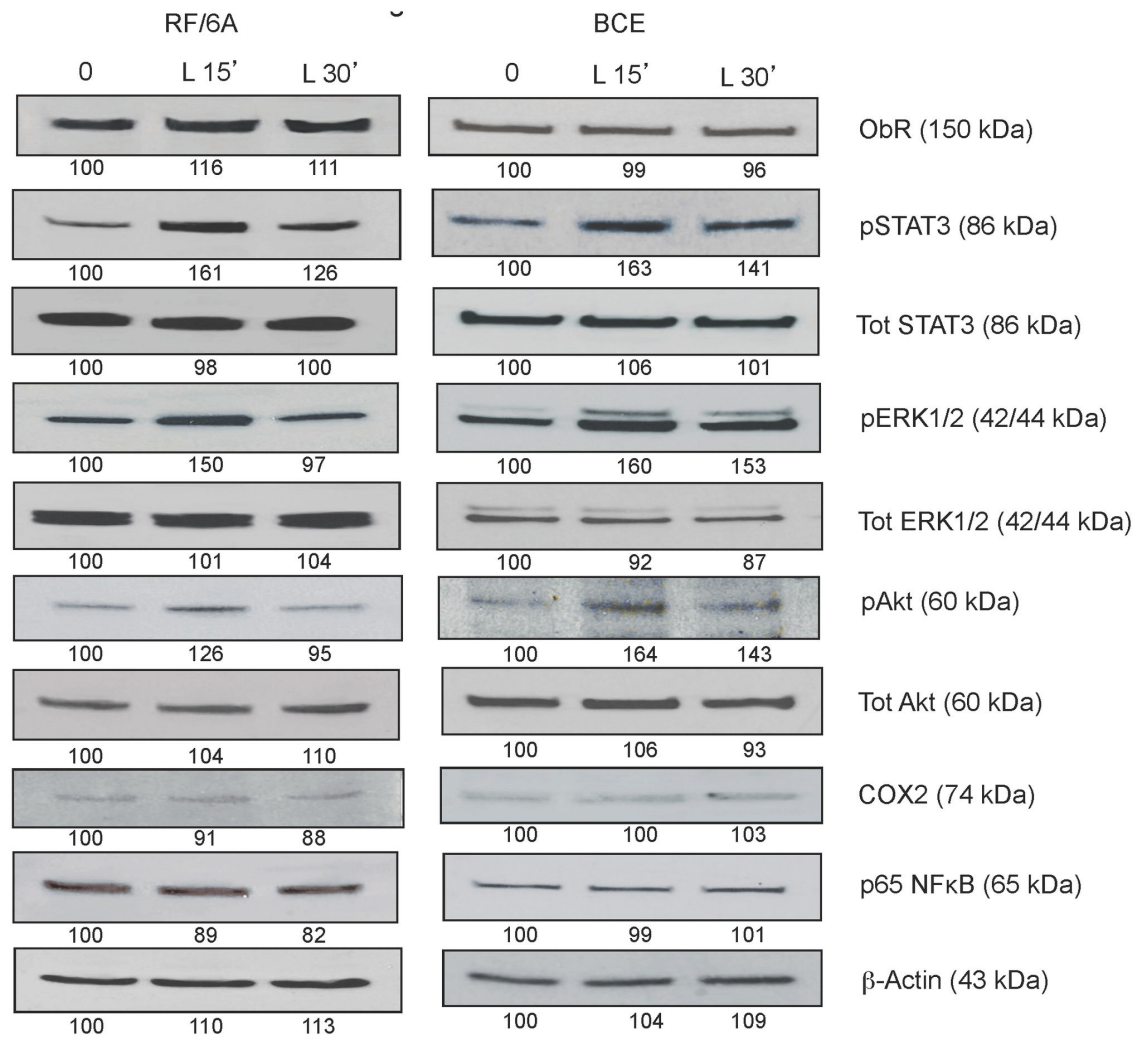
Leptin effects on intracellular signaling in RF/6A and BCE cells. RF/6A and BCE cells were stimulated with 250 ng/mL leptin (L) for 15 and 30 min and the expression of phosphorylated (p) and total (Tot) proteins was assessed by WB and quantified as described in Materials and Methods. The levels of β-actin were assessed as control of loading. The numbers under WB panels represent relative densitometry values (%) of phosphorylated and total proteins, with the value in SFM is taken as 100%. The representative blots of at least 3 experiments are shown.

Leptin at 250 ng/mL activated STAT3, ERK1/2, and Akt in both cell lines at 15 min. Specifically, the phosphorylation of STAT3 was upregulated by 64 and 58%, of ERK1/2 by 65 and 49%, and of Akt by 21 and 55% in RF/6A and BCE, respectively. The response to leptin was in most cases less pronounced at 30 min (Figure 5). The short leptin exposure did not affect the expression of p65 NFκB and COX2 (the latter was barely detectable in BCE cells) (Figure 5).

**Figure 6:**
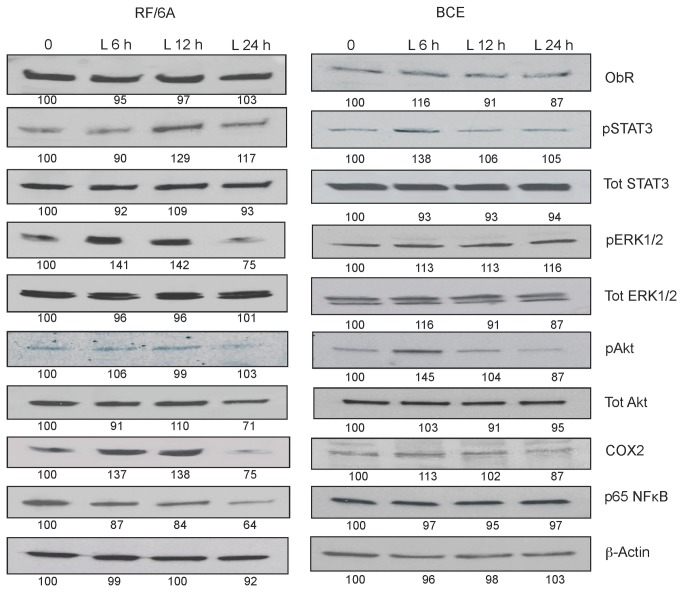
Long-term intracellular leptin effects in RF/6A and BCE cells. RF/6A and BCE cells were stimulated with 250 ng/mL leptin (L) for 6, 12, 24 h as described in Materials and Methods. The numbers under WB panels represent relative densitometry values (%) of phosphorylated and total proteins, with the value in SFM is taken as 100%. The representative blots of at least 3 experiments are shown.

**Figure 7:**
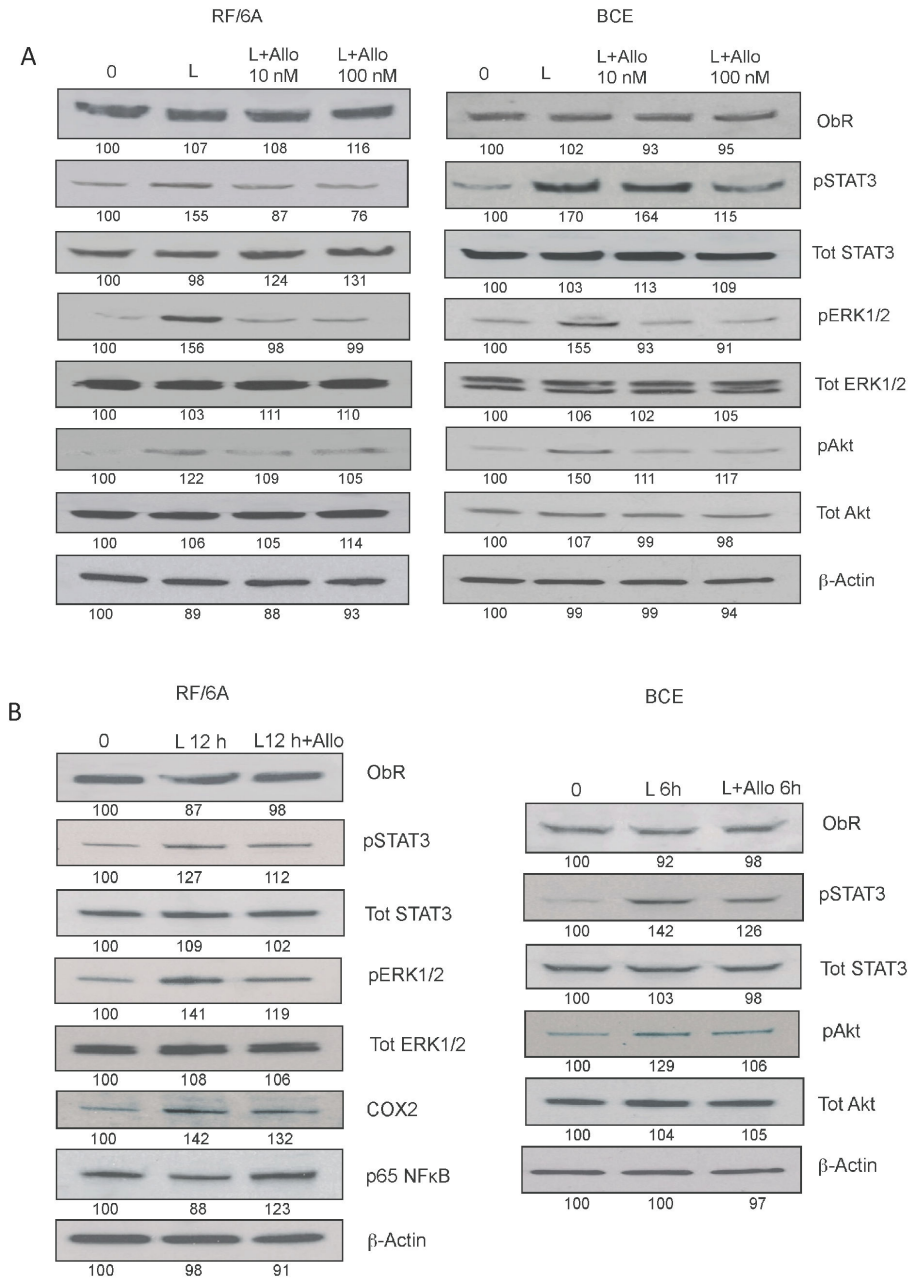
Effects of Allo-aca on intracellular leptin effects in RF/6A and BCE cells. **A**. The effects of 10 and 100 nM Allo-aca (Allo) on acute intracellular signaling were tested in RF/6A and BCE cells stimulated with 250 ng/mL leptin (L) for 30 min. **B**. The long-term effects of 100 nM Allo-aca (Allo) on intracellular pathways were assessed in cells stimulated for 12 h (RF/6A) or 6 h (BCE) with 250 ng/mL leptin (L) and measured as described under Figures 5 and 6. The representative blots of at least 3 experiments are shown.

We also studied long-term effects of leptin stimulation on the expression and/or activation of major ObR downstream targets (Figure 6). In general, the maximal responses were noted at 6-12 h in RF/6A cells and at 6 h in BCE cells. In RF/6A cells, a prolonged exposure to 250 ng/mL leptin increased the expression of COX2 by ~38%, decreased the expression of NFκB by 16% as well as upregulated STAT3, ERK1/2 phosphorylation by 18% and 48%, respectively. In BCE cells, 6 h leptin stimulation did not modulate COX2 or NFκB, but increased STAT3 and Akt phosphorylation by 48 and 40%, respectively (Figure 6). Interestingly, 24 h leptin treatment decreased COX2 and NFκB expression in RF/6A cells by 20% and 30%, respectively. The above intracellular responses were significantly reduced in the presence of 100 nM Allo-aca (Figure 7). None of the stimulatory or inhibitory treatments altered the expression of ObR in studied cells (Figures 5-7).

### Leptin upregulates leptin mRNA and protein expression in RF/6A and BCE cells. Allo-aca antagonizes this effect.

**Figure 8:**
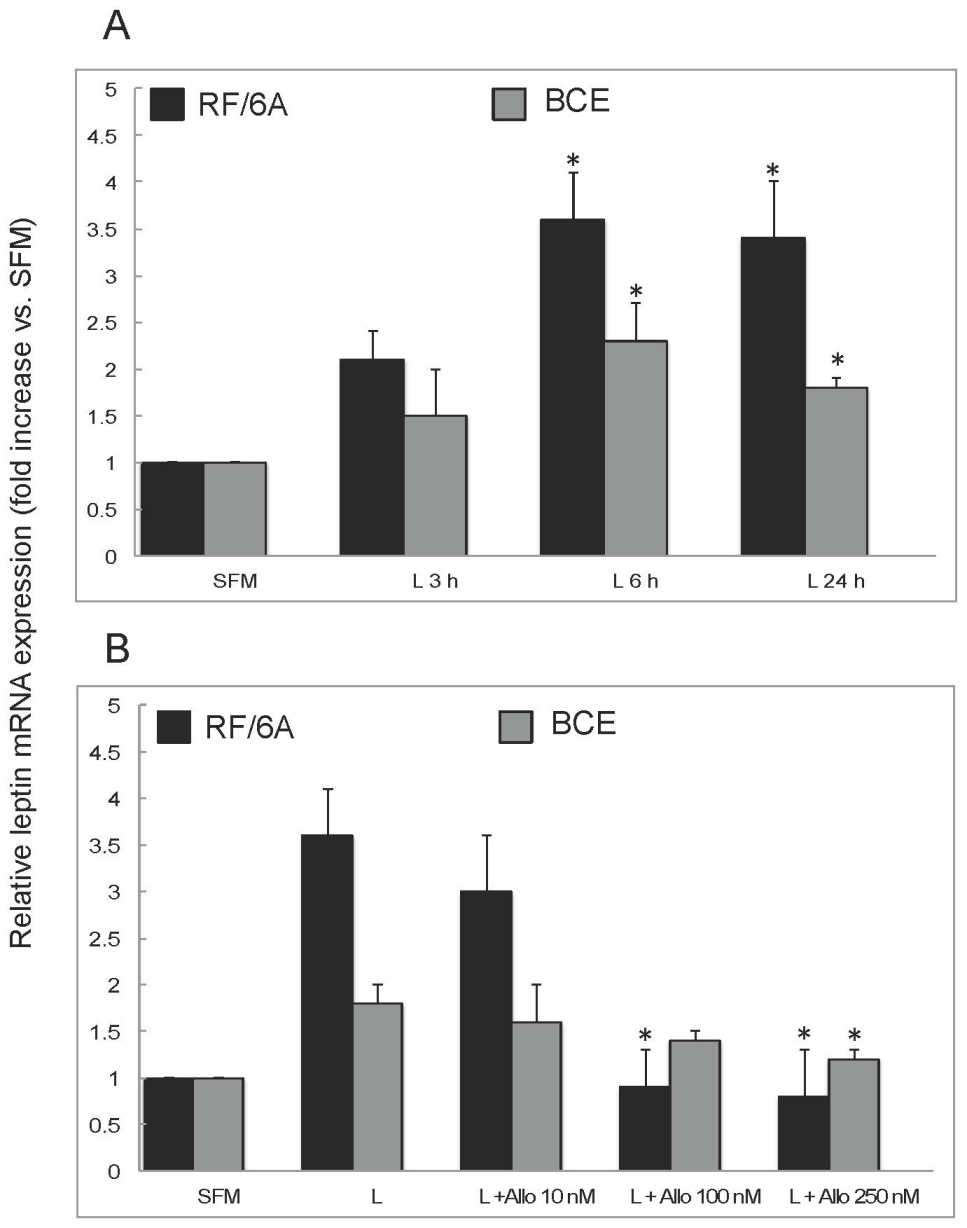
Effects of leptin and Allo-aca on leptin mRNA expression in RF/6A and BCE cells. **A**. RF/6A and BCE cells were stimulated with 250 ng/mL leptin (L) for 3, 6, and 24 h. The expression of leptin mRNA was assessed by QRT-PCR as described in Materials and Methods. The values represent fold increase (± SD) of leptin mRNA levels in leptin treated cells vs. untreated controls (C=SFM) assigned value 1. **B**. The cells were pretreated with 10, 100, or 250 nM Allo-aca (Allo) for 1 h and then stimulated with 250 ng/mL leptin (L) for 6 h. Leptin mRNA was measured by QRT-PCR. Asterisks indicate significant (p≤0.05) differences vs. SFM (**A**) or leptin (**B**).

**Figure 9:**
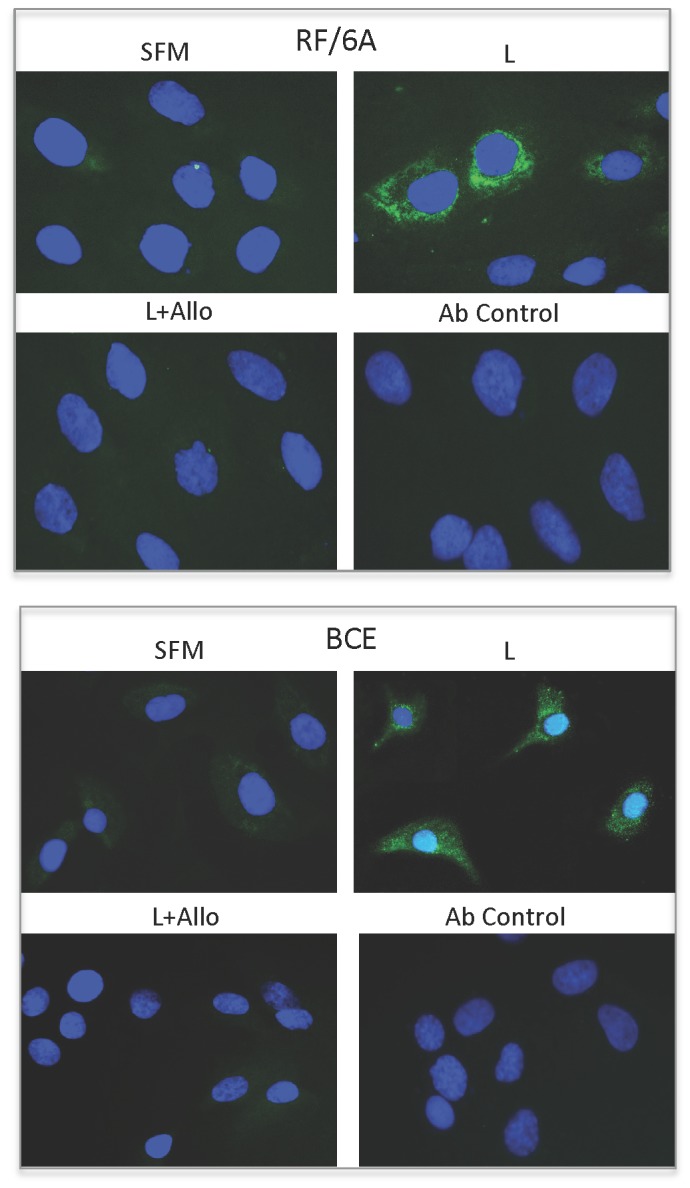
Effects of leptin and Allo-aca on leptin protein expression in RF/6A and BCE cells. RF/6A and BCE cells were synchronized in SFM and stimulated with 250 ng/mL leptin (L) for 24 h in the presence or absence of 100 nM (for RF/6A) or 250 nM (for BCE) Allo-aca (Allo). The expression of leptin protein (green immunofluorescence) in treated and untreated cells was detected with specific Abs, as described in Materials and Methods. In control experiments, the primary Ab was omitted.

We found that under basal growth conditions RF6A and BCE cells synthesize leptin mRNA, the latter expressing ~7-fold more than the former (data not shown). The treatment with exogenous leptin for 3, 6, and 24 h further potentiated leptin mRNA expression in both cell lines. The maximal effects of leptin on its own mRNA synthesis, i.e., ~3.6-fold increase in RF/6A at 6-24 h and 1.7-fold increase in BCE cells at 6 h of stimulation (Figure 8A). This autocrine leptin synthesis was abolished by 100-250 nM Allo-aca in RF/6A cells and reduced by ~33% in the presence of 250 nM in BCE cells (Figure 8B). Similarly, we found that both cell lines express endogenous leptin protein. In BCE cultures, ~35% of cells expressed leptin under SFM conditions, while in RF/6A cultures, only ~ 6% of cells were strongly positive for leptin immunostaining. Treatment with leptin increased the number of leptin expressing cells by 60% in RF/6A cultures and by 30% in BCE cells. The increased leptin expression was blocked with 100 and 250 nM Allo-aca in RF/6A and BCE cells, respectively (Figure 9).

## Discussion

Ocular neoangiogenesis is one of the mechanisms implicated in vision threatening diseases, such as PDR, AMD and some corneal pathologies [2]. Current treatments for these diseases include laser photocoagulation, vitrectomy surgery and/or intravitreal injections of anti-VEGF agents. At present, FDA-approved treatments for wet AMD and DME include modified anti-VEGF antibodies and VEGF trap proteins. These drugs are experimentally used for other eye diseases, e.g., PDR [7].

In addition to VEGF and VEGFR, other mediators of ocular neovascularization are being considered as potential therapeutic targets [4]. This includes leptin, an upstream activator of VEGF pathways as well as an independent angiogenic and inflammatory agent [6,21].

Although leptin is known to promote angiogenesis and endothelial cell growth [18,21,22,43], its function in ophthalmic cell models has not been systematically explored. Only one study described leptin effects in porcine retinal endothelial cells and provided a very limited data on ObR signaling [24]. Similarly, targeting ObR in ophthalmic in vitro or in vivo models has never been attempted.

Here, we studied biological effects of leptin and consequences of interfering with leptin signals in RF/6A retinal and BCE corneal cells that are accepted preclinical ophthalmic in vitro models [44-50]. In both cell lines, leptin stimulated mitogenesis at 24-72 h at 50-250 ng/mL concentrations. The observed ~30-35% growth response in response to 50-250 ng/mL leptin treatment in RF/6A and BCE cells is consistent with data obtained in non-ophthalmic endothelial cells [18,19,21-23,51] and other cell types [41,52]. Notably, the mitogenic concentrations of leptin in RF/6A and BCE cells were in the upper range of vitreous leptin levels reported in PDR patients (~37 ng/mL) [32].

In this report, we demonstrated that biological leptin effects were paralleled by the activation of several acute and long-term responses. Intracellular leptin signaling in RF/6A and BCE cells was in part similar to that described for other cell types [14,21,27,34,41,53] but also exhibited some unique features. In both cell lines, short-term leptin exposure activated the STAT3, Akt, and ERK1/2 pathways but did not modulate the expression of the inflammatory mediators COX2 and p65 NFκB. In contrast, more variability was noted under long-term leptin exposure. In both cell lines, 6 or 12 h leptin stimulation increased STAT3 phosphorylation. The treatment also augmented ERK1/2 activation at 6-12 h in RF/6A, but not in BCE cells. On the other hand, the activation of Akt was seen only in BCE cells at 6 h. The reason for these variations in signaling dynamics is unknown. It might reflect the levels of signaling molecules as well as dynamics in endogenous leptin synthesis.

Garonna et al. described that prolonged leptin exposure upregulated COX2 expression in HUVEC [21]. Similarly, we noted significantly increased COX2 expression at 6 and 12 h in RF/6A cells, which was followed by a decrease in COX2 content at 24 h. However, in BCE cells, expressing very low COX2 levels, leptin did not affect this protein in a significant manner. We also noted cell-specific leptin effects on the NFκB pathway. Specifically, we and others described in various non-ophthalmic models that leptin can stimulate NFκB through upregulation of p65 (Rel A) phosphorylation [37,53-55]. In this study, however, we found a progressive decrease of p65 NFκB levels at 6-12 h of leptin exposure in RF/6A cells. This was somewhat surprising as coordinate expression of NFκB and COX2 was described in RF/6A cells in response to hypoxic conditions, and NFκB activation appeared to precede COX2 expression [56]. Potentially, temporal p 65 NFκB downregulation could represent natural oscillation of this factor [57]. One study described activation of p38 kinase in response to leptin treatment in HUVEC [21], however, p38 levels were very low in both our cell lines and we were not able to detect any regulation of this kinase upon leptin short- or long-term leptin treatment (data not shown).

Interestingly, we found that the presence of leptin augments its own mRNA and protein synthesis in both cell lines. While leptin protein was found in ocular tissues by immunohistochemistry and ELISA [31-33,58], we are the first to report that retinal and corneal endothelial cells can regulate leptin expression through an autocrine mechanism. The possibility of intraocular leptin synthesis is also suggested by our preliminary findings that leptin mRNA is expressed in the eyes of animals with laser-induced neovascularization (data not shown). This suggests that retinal and corneal endothelial cells can produce endogenous leptin as well as respond to leptin in the circulation. Indeed, local and systemic leptin sources in ocular disease were previously suggested based on data obtained in patients [31,32]. In this context, hyperleptinemia associated with diabetes or obesity might influence ocular neovascularization in the situations of compromised blood-retinal barrier.

All of the biological effects of leptin in RF/6A and BCE cells were inhibited by a specific ObR antagonist, Allo-aca. The efficacy of Allo-aca in endothelial cell models has never been tested before and our present data represent the first original report on targeting ObR in ophthalmic cells. In particular, Allo-aca reduced RF/6A and BCE cell growth to basal levels at 100 nM, tube formation at 50-100 nM, cell signaling at 100 nM, and leptin mRNA and protein expression at 100-250 nM concentrations.

In summary, our report provides the original evidence that targeting ObR can reduce leptin mitogenesis and angiogenic differentiation in two different ophthalmic cell lines. Taking into consideration crosstalk between leptin and VEGF systems, one could envision that treatments employing combinations of drugs targeting both pathways could offer better efficacy and limit drug side effects and development of resistance.

## References

[1] Chang JH, Garg NK, Lunde E, Han KY, Jain S et al. (2012) Corneal neovascularization: an anti-VEGF therapy review. Surv Ophthalmol 57: 415-429. doi:10.1016/j.survophthal.2012.01.007. PubMed: 22898649. 22898649 PMC3709023

[2] Lee P, Wang CC, Adamis AP (1998) Ocular neovascularization: an epidemiologic review. Surv Ophthalmol 43: 245-269. doi:10.1016/S0039-6257(98)00035-6. PubMed: 9862312. 9862312

[3] Qazi Y, Maddula S, Ambati BK (2009) Mediators of ocular angiogenesis. J Genet 88: 495-515. doi:10.1007/s12041-009-0068-0. PubMed: 20090210. 20090210 PMC3306772

[4] Tang J, Kern TS (2011) Inflammation in diabetic retinopathy. Prog Retin Eye Res 30: 343-358. doi:10.1016/j.preteyeres.2011.05.002. PubMed: 21635964. 21635964 PMC3433044

[5] Misra GP, Imai H, Singh RJ, Lowe TL, Gardner TW (2010) Diabetic retinopathy and diabetic macular edema. In: Sauders WB Edinburgh: Retinal Pharmacology: Elsevir. pp. 133-136

[6] Praidou A, Androudi S, Brazitikos P, Karakiulakis G, Papakonstantinou E et al. (2010) Angiogenic growth factors and their inhibitors in diabetic retinopathy. Curr. Diabetes Rev 6: 304-312. doi:10.2174/157339910793360815. 20594164

[7] Willard AL, Herman IM (2012) Vascular complications and diabetes: current therapies and future challenges. J Ophthalmol, 2012: 2012: 209538. PubMed: 22272370 10.1155/2012/209538 PMC3261480 22272370

[8] Stewart MW (2012) The expanding role of vascular endothelial growth factor inhibitors in ophthalmology. Mayo Clin Proc 87: 77-88. doi:10.1016/j.mayocp.2012.08.003. PubMed: 22212972. 22212972 PMC3498409

[9] Truong A, Wong TY, Khachigian LM (2011) Emerging therapeutic approaches in the management of retinal angiogenesis and edema. J Mol Med (Berl) 89: 343-361. doi:10.1007/s00109-010-0709-z. PubMed: 21170513. 21170513

[10] Friedman JM, Halaas JL (1998) Leptin and the regulation of body weight in mammals. Nature 395: 763-770. doi:10.1038/27376. PubMed: 9796811. 9796811

[11] Wauters M, Considine RV, Van Gaal LF (2000) Human leptin: from an adipocyte hormone to an endocrine mediator. Eur J Endocrinol 143: 293-311. doi:10.1530/eje.0.1430293. PubMed: 11022169. 11022169

[12] Sweeney G (2010) Cardiovascular effects of leptin. Nat. Rev Cardiol 7: 22-29. doi:10.1038/nrcardio.2009.224. 19949425

[13] Zhang F, Chen Y, Heiman M, Dimarchi R (2005) Leptin: structure, function and biology. Vitam Horm 71: 345-372. doi:10.1016/S0083-6729(05)71012-8. PubMed: 16112274. 16112274

[14] Garofalo C, Surmacz E (2006) Leptin and cancer. J Cell Physiol 207: 12-22. doi:10.1002/jcp.20472. PubMed: 16110483. 16110483

[15] Scolaro L, Cassone M, Kolaczynski JW, Otvos L Jr, Surmacz E (2010) Leptin-based therapeutics. Expert. Rev Endocrinol Metab 5: 875-889. doi:10.1586/eem.10.61. 30780830

[16] Margetic S, Gazzola C, Pegg GG, Hill RA (2002) Leptin: a review of its peripheral actions and interactions. Int J Obes Relat Metab Disord 26: 1407-1433. doi:10.1038/sj.ijo.0802142. PubMed: 12439643. 12439643

[17] Anagnostoulis S, Karayiannakis AJ, Lambropoulou M, Efthimiadou A, Polychronidis A et al. (2008) Human leptin induces angiogenesis in vivo. Cytokine 42: 353-357. doi:10.1016/j.cyto.2008.03.009. PubMed: 18448353. 18448353

[18] Bouloumié A, Drexler HC, Lafontan M, Busse R (1998) Leptin, the product of Ob gene, promotes angiogenesis. Circ Res 83: 1059-1066. doi:10.1161/01.RES.83.10.1059. PubMed: 9815153. 9815153

[19] Park HY, Kwon HM, Lim HJ, Hong BK, Lee JY et al. (2001) Potential role of leptin in angiogenesis: leptin induces endothelial cell proliferation and expression of matrix metalloproteinases in vivo and in vitro. Exp Mol Med 33: 95-102. doi:10.1038/emm.2001.17. PubMed: 11460888. 11460888

[20] Sierra-Honigmann MR, Nath AK, Murakami C, García-Cardeña G, Papapetropoulos A et al. (1998) Biological action of leptin as an angiogenic factor. Science 281: 1683-1686. doi:10.1126/science.281.5383.1683. PubMed: 9733517. 9733517

[21] Garonna E, Botham KM, Birdsey GM, Randi AM, Gonzalez-Perez RR et al. (2011) Vascular endothelial growth factor receptor-2 couples cyclo-oxygenase-2 with pro-angiogenic actions of leptin on human endothelial cells. PLOS ONE 6: e18823. doi:10.1371/journal.pone.0018823. PubMed: 21533119. 21533119 PMC3078934

[22] Ferla R, Bonomi M, Otvos L Jr., Surmacz E (2011) Glioblastoma-derived leptin induces tube formation and growth of endothelial cells: comparison with VEGF effects. BMC Cancer 11: 303. doi:10.1186/1471-2407-11-303. PubMed: 21771332. 21771332 PMC3146945

[23] Cao R, Brakenhielm E, Wahlestedt C, Thyberg J, Cao Y (2001) Leptin induces vascular permeability and synergistically stimulates angiogenesis with FGF-2 and VEGF. Proc Natl Acad Sci U S A 98: 6390-6395. doi:10.1073/pnas.101564798. PubMed: 11344271. 11344271 PMC33478

[24] Suganami E, Takagi H, Ohashi H, Suzuma K, Suzuma I et al. (2004) Leptin stimulates ischemia-induced retinal neovascularization: possible role of vascular endothelial growth factor expressed in retinal endothelial cells. Diabetes 53: 2443-2448. doi:10.2337/diabetes.53.9.2443. PubMed: 15331557. 15331557

[25] Bjørbaek C, Buchholz RM, Davis SM, Bates SH, Pierroz DD et al. (2001) Divergent roles of SHP-2 in ERK activation by leptin receptors. J Biol Chem 276: 4747-4755. doi:10.1074/jbc.M007439200. PubMed: 11085989. 11085989

[26] Villanueva EC, Myers MG Jr. (2008) Leptin receptor signaling and the regulation of mammalian physiology. Int J Obes (Lond) 32 Suppl 7: S8-12. doi:10.1038/ijo.2008.82. PubMed: 19136996. PMC2648306 19136996

[27] Sweeney G (2002) Leptin signalling. Cell Signal 14: 655-663. doi:10.1016/S0898-6568(02)00006-2. PubMed: 12020765. 12020765

[28] Zabeau L, Lavens D, Peelman F, Eyckerman S, Vandekerckhove J et al. (2003) The ins and outs of leptin receptor activation. FEBS Lett 546: 45-50. doi:10.1016/S0014-5793(03)00440-X. PubMed: 12829235. 12829235

[29] Basak S, Duttaroy AK (2012) Leptin induces tube formation in first-trimester extravillous trophoblast cells. Eur J Obstet Gynecol Reprod Biol 164: 24-29. doi:10.1016/j.ejogrb.2012.05.033. PubMed: 22717511. 22717511

[30] Sun G, Su G, Zhang M, Zeng Q, Shi Q (2011) Effect of tetrandrine on the expression of leptin (LP) and vascular endothelial growth factor (VEGF) in corneal neovascularization of rats. Scient Res Essays 6: 5008-5013.

[31] Gariano RF, Nath AK, D’Amico DJ, Lee T, Sierra-Honigmann MR (2000) Elevation of vitreous leptin in diabetic retinopathy and retinal detachment. Invest Ophthalmol Vis Sci 41: 3576-3581. PubMed: 11006255. 11006255

[32] Maberley D, Cui JZ, Matsubara JA (2006) Vitreous leptin levels in retinal disease. Eye (Lond) 20: 801-804. doi:10.1038/sj.eye.6702011. PubMed: 16052255. 16052255

[33] Hernández C, Lecube A, Castellanos JM, Segura RM, Garat M et al. (2004) Intravitreous leptin concentrations in patients with proliferative diabetic retinopathy. Retina 24: 30-35. doi:10.1097/00006982-200402000-00005. PubMed: 15076941. 15076941

[34] Beccari S, Kovalszky I, Wade JD, Otvos L, Surmacz E (2013) Designer peptide antagonist of the leptin receptor with peripheral antineoplastic activity. Peptides (. (2013)) PubMed: 23567149. 10.1016/j.peptides.2013.03.027 23567149

[35] Otvos L Jr, Kovalszky I, Scolaro L, Sztodola A, Olah J et al. (2011) Peptide-based leptin receptor antagonists for cancer treatment and appetite regulation. Biopolymers 96: 117-125. doi:10.1002/bip.21377. PubMed: 20564005. 20564005

[36] Kovalszky I, Surmacz E, Scolaro L, Cassone M, Ferla R et al. (2010) Leptin-based glycopeptide induces weight loss and simultaneously restores fertility in animal models. Diabetes Obes Metab 12: 393-402. doi:10.1111/j.1463-1326.2009.01170.x. PubMed: 20415687. 20415687

[37] Otvos L Jr., Shao WH, Vanniasinghe AS, Amon MA, Holub MC et al. (2011) Toward understanding the role of leptin and leptin receptor antagonism in preclinical models of rheumatoid arthritis. Peptides 32: 1567-1574. doi:10.1016/j.peptides.2011.06.015. PubMed: 21723351. 21723351

[38] Otvos L Jr., Cassone M, Terrasi M, Cascio S, Mateo GD et al. (2009) Agonists and partial antagonists acting on the leptin--leptin receptor interface. Adv Exp Med Biol 611: 497-498. doi:10.1007/978-0-387-73657-0_215. PubMed: 19400282. 19400282

[39] Otvos L Jr., Kovalszky I, Riolfi M, Ferla R, Olah J et al. (2011) Efficacy of a leptin receptor antagonist peptide in a mouse model of triple-negative breast cancer. Eur J Cancer 47: 1578-1584. doi:10.1016/j.ejca.2011.01.018. PubMed: 21353530. 21353530

[40] Otvos L Jr., Surmacz E (2011) Targeting the leptin receptor: a potential new mode of treatment for breast cancer. Expert Rev Anticancer Ther 11: 1147-1150. doi:10.1586/era.11.109. PubMed: 21916566. 21916566

[41] Garofalo C, Sisci D, Surmacz E (2004) Leptin interferes with the effects of the antiestrogen ICI 182,780 in MCF-7 breast cancer cells. Clin Cancer Res 10: 6466-6475. doi:10.1158/1078-0432.CCR-04-0203. PubMed: 15475434. 15475434

[42] Cascio S, Bartella V, Auriemma A, Johannes GJ, Russo A et al. (2008) Mechanism of leptin expression in breast cancer cells: role of hypoxia-inducible factor-1alpha. Oncogene 27: 540-547. doi:10.1038/sj.onc.1210660. PubMed: 17653093. 17653093

[43] Gonzalez RR, Cherfils S, Escobar M, Yoo JH, Carino C et al. (2006) Leptin signaling promotes the growth of mammary tumors and increases the expression of vascular endothelial growth factor (VEGF) and its receptor type two (VEGF-R2). J Biol Chem 281: 26320-26328. doi:10.1074/jbc.M601991200. PubMed: 16825198. 16825198

[44] Balaiya S, Khetpal V, Chalam KV (2012) Hypoxia initiates sirtuin1-mediated vascular endothelial growth factor activation in choroidal endothelial cells through hypoxia inducible factor-2alpha. Mol Vis 18: 114-120. PubMed: 22275802. 22275802 PMC3265172

[45] Dong X, Wang YS, Dou GR, Hou HY, Shi YY et al. (2011) Influence of Dll4 via HIF-1alpha-VEGF signaling on the angiogenesis of choroidal neovascularization under hypoxic conditions. PLOS ONE 6: e18481. doi:10.1371/journal.pone.0018481. PubMed: 21526177. 21526177 PMC3079714

[46] Huang L, Yu W, Li X, Xu Y, Niu L et al. (2009) Expression of Robo4 in the fibrovascular membranes from patients with proliferative diabetic retinopathy and its role in RF/6A and RPE cells. Mol Vis 15: 1057-1069. PubMed: 19495426. 19495426 PMC2689875

[47] Ottino P, Finley J, Rojo E, Ottlecz A, Lambrou GN et al. (2004) Hypoxia activates matrix metalloproteinase expression and the VEGF system in monkey choroid-retinal endothelial cells: Involvement of cytosolic phospholipase A2 activity. Mol Vis 10: 341-350. PubMed: 15162095. 15162095

[48] Xu Y, Zhao H, Zheng Y, Gu Q, Ma J et al. (2010) A novel antiangiogenic peptide derived from hepatocyte growth factor inhibits neovascularization in vitro and in vivo. Mol Vis 16: 1982-1995. PubMed: 21031024. 21031024 PMC2956696

[49] Rusovici R, Sakhalkar M, Chalam KV (2011) Evaluation of cytotoxicity of bevacizumab on VEGF-enriched corneal endothelial cells. Mol Vis 17: 3339-3346. PubMed: 22219629. 22219629 PMC3247162

[50] Rusovici R, Patel CJ, Chalam KV (2013) Bevacizumab inhibits proliferation of choroidal endothelial cells by regulation of the cell cycle. Clin. Ophthalmologe 7: 321-327. 10.2147/OPTH.S41556 PMC3575188 23430458

[51] Artwohl M, Roden M, Hölzenbein T, Freudenthaler A, Waldhäusl W et al. (2002) Modulation by leptin of proliferation and apoptosis in vascular endothelial cells. Int J Obes Relat Metab Disord 26: 577-580. doi:10.1038/sj.ijo.0801947. PubMed: 12075587. 12075587

[52] Oda A, Taniguchi T, Yokoyama M (2001) Leptin stimulates rat aortic smooth muscle cell proliferation and migration. Kobe J Med Sci 47: 141-150. PubMed: 11729375. 11729375

[53] Gonzalez-Perez RR, Xu Y, Guo S, Watters A, Zhou W et al. (2010) Leptin upregulates VEGF in breast cancer via canonic and non-canonical signalling pathways and NFkappaB/HIF-1alpha activation. Cell Signal 22: 1350-1362. doi:10.1016/j.cellsig.2010.05.003. PubMed: 20466060. 20466060 PMC2928711

[54] Rouet-Benzineb P, Aparicio T, Guilmeau S, Pouzet C, Descatoire V et al. (2004) Leptin counteracts sodium butyrate-induced apoptosis in human colon cancer HT-29 cells via NF-kappaB signaling. J Biol Chem 279: 16495-16502. doi:10.1074/jbc.M312999200. PubMed: 14752104. 14752104

[55] Yeh WL, Lu DY, Lee MJ, Fu WM (2009) Leptin induces migration and invasion of glioma cells through MMP-13 production. Glia 57: 454-464. doi:10.1002/glia.20773. PubMed: 18814267. 18814267

[56] Lukiw WJ, Ottlecz A, Lambrou G, Grueninger M, Finley J et al. (2003) Coordinate activation of HIF-1 and NF-kappaB DNA binding and COX-2 and VEGF expression in retinal cells by hypoxia. Invest Ophthalmol Vis Sci 44: 4163-4170. doi:10.1167/iovs.02-0655. PubMed: 14507857. 14507857

[57] Nelson DE, Ihekwaba AE, Elliott M, Johnson JR, Gibney CA et al. (2004) Oscillations in NF-kappaB signaling control the dynamics of gene expression. Science 306: 704-708. doi:10.1126/science.1099962. PubMed: 15499023. 15499023

[58] Ricker LJ, Kijlstra A, Kessels AG, de Jager W, Hendrikse F et al. (2012) Adipokine levels in subretinal fluid from patients with rhegmatogenous retinal detachment. Exp Eye Res 94: 56-62. doi:10.1016/j.exer.2011.11.006. PubMed: 22138416. 22138416

